# Sex and age differences in the achievement of control targets in patients with type 2 diabetes: results from a population-based study in a South European region

**DOI:** 10.1186/s12875-016-0533-9

**Published:** 2016-10-12

**Authors:** K. Cambra, A. Galbete, L. Forga, O. Lecea, M. J. Ariz, C. Moreno-Iribas, F. Aizpuru, B. Ibañez

**Affiliations:** 1 Navarrabiomed-FMS, C/ Irunlarrea 8, Recinto CHN, 31008 Pamplona, Spain; 2 Health Services Research on Chronic Patients Network (REDISSEC), Pamplona, Spain; 3 IdiSNA, Pamplona, Spain; 4 Complejo Hospitalario de Navarra, Servicio Navarro de Salud-Osasunbidea, Pamplona, Spain; 5 Gerencia de Atención Primaria, Servicio Navarro de Salud-Osasunbidea, Pamplona, Spain; 6 Instituto de Salud Pública y Laboral de Navarra, Pamplona, Spain; 7 Hospital de Txagorritxu, Servicio Vasco de Salud-Osakidetza, Vitoria Gasteiz, Spain

**Keywords:** Type 2 diabetes, Control targets, Health inequalities, Glycemic control, Cardiovascular risk factors

## Abstract

**Background:**

We aimed to determine the degree to which control targets of glycaemia and cardiovascular risk factors were achieved among patients with type 2 diabetes and to investigate sex- and age-related differences in this population.

**Methods:**

This cross-sectional, population-based study was conducted in Spain. Glycated hemoglobin (HbA1c), blood pressure, LDL-c, HDL-c, triglycerides, BMI, and smoking history were obtained from electronic clinical primary care records (*n* = 32,638 cases). The proportions of patients who met control targets were determined according to sex and age groups. Comparisons between groups were conducted with t-tests for continuous variables, tests for trends in proportions for categorical and ordinal variables, and Pearson’s chi-square tests and binary logistic regression models for categorical variables.

**Results:**

The overall proportions of patients with type 2 diabetes who met the target objectives for HbA1c (<7 %, 53 mmol/mol), blood pressure (130/80 mmHg), and LDL-cholesterol (100 mg/dl) were 60, 40 and 41 %, respectively. Women were less likely than men to meet the control targets of HbA1c (59 vs 61 %), LDL (35 vs 45 %), and HDL (58 vs 78 %). Patients under 65 years of age presented poorer control than older age groups. Only a minority of patients with type 2 diabetes met the composite target objectives for glycemic control, blood pressure, and LDL.

**Conclusions:**

There are differential gaps in the control results of female patients and younger patients, which should prompt improvements in case management and care. There is room for further improvement in the cardiometabolic control of patients with type 2 diabetes.

**Electronic supplementary material:**

The online version of this article (doi:10.1186/s12875-016-0533-9) contains supplementary material, which is available to authorized users.

## Background

In 2015, there were an estimated 415 million diabetics worldwide, corresponding to 8.3 % of the population. This figure is estimated to increase to 642 million by 2040 [1]. The di@betes study in Spain found that 13.8 % of the population over 18 years of age had type 2 diabetes, and almost half of those cases (6 %) were undiagnosed [2].

Chronic complications of diabetes impose morbidities that reduce life quality and expectancy. The Diabetes Control and Complication Trial (DCCT) and the United Kingdom Prospective Diabetes Study (UKPDS) showed that improved glycemic control was associated with a reduction in the risk of complications in type 1 and type 2 diabetics, respectively, and subsequent studies have shown that patients with diabetes can largely reduce the risk of cardiovascular disease by lowering blood pressure and LDL-cholesterol levels and avoiding tobacco consumption [3–8]. Accordingly, there has been a broad consensus in recent years to develop and extensively implement clinical practice guidelines for diabetes care and treatment [9–12].

However, despite the scientific evidence and the high degree of consensus reached by experts, clinicians, and planners, achieving control targets remains a challenge. Several studies have shown that there is an important gap between recommendations and clinical practice, and only a minority of diabetic patients achieve the optimal control of glycaemia and cardiovascular risk factors [13–18].

Electronic information systems that maintain updated information on every contact with patients are promising sources of information, particularly for the identification of health needs and the evaluation of interventions, because they can provide clinical practice information in real conditions. Thus, electronic information systems may help characterize which diabetic patients achieve or do not achieve control targets and may help identify the factors that determine those achievements, both of which are key issues to optimize in intervention strategies.

This population-based study used existing electronic medical primary care records. The aims of this study were to assess the actual degree of achievement of glycemic control and cardiovascular risk factors and to investigate differences by sex and age among patients with type 2 diabetes.

## Methods

This cross-sectional study was conducted in Navarra, a community in northern Spain with over 600,000 inhabitants. In Navarra, Primary Care was developed in the 1980s and provides universal health coverage to the population. The territory is divided into 57 Basic Zones, each of which contains a team of general practitioners, nurses, and pediatricians who belong to the Regional Health Service of Navarre (RHSN). The populations of the Basic Zones range from 5000 inhabitants in rural areas to 25,000 in urban areas. The RHSN is essentially financed by general taxes, and health services, hospitalizations, and diagnostic procedures are free of charge for all citizens. A fraction of the medication cost is paid by patients, depending on their work status and income. Antidiabetic drugs are subsidized at least by 90 %.

Within the RHSN, all patients with type 2 diabetes are managed by primary care teams. If the patients do not achieve an acceptable glycemic control they are derived to the ambulatory specialized care and then attended by endocrinologists. Also, they are treated by specialists when they are hospitalized or have complications. Thus, the Primary Care Electronic Medical Record System, named Atenea, contains information related to all the patients with type 2 diabetes managed by the RHSN. Specifically, it contains data about diagnoses, clinical variables, life styles, laboratory results, and prescriptions. Primary care electronic medical records were established in Navarra in the early 2000s and have been thoroughly used by all professionals since 2008. Electronic prescriptions were established in 2013.

### Study population and selection of study variables

The study population comprised all of the patients who were registered in Atenea with a diagnosis of type 2 diabetes (International Classification of Primary Care (ICPC), T90) on May 15^th^, 2014. We obtained the following patient information: glycated hemoglobin (HbA1c), systolic and diastolic blood pressure (SBP and DBP), LDL- and HDL- cholesterol levels, triglycerides, weight, height, body mass index (BMI), smoking history, medications, date of registration in the information system, date of diabetic onset, and birth date. We obtained the latest results available within the 15 months prior to the data extraction date. We provide the number of data entries available for each of the study variables. Missing values imply that the patients had not had controls performed within the previous 15 months, and they can be regarded as a negative indicator of the process of care.

### Statistical analysis

The continuous variables were summarized by the mean and standard deviation; the categorical variables were summarized by frequencies. Comparisons by gender and age groups were conducted with t-tests for continuous variables, tests for trends in proportions for ordinal variables, and Pearson’s chi-square tests for categorical variables. The differences in the degree of achievement of control targets between men and women were assessed by binary logistic regression models, which included age and the duration of diabetes as covariates. The statistical analyses were conducted with IMB-SPSS 20 and R 3.2.0.

## Results

The total number of registered Navarra Regional Health Service patients over 20 years of age with type 2 diabetes was 32,638. There were 18,188 men and 14,018 women with diabetes, which yielded an overall prevalence of 6.6 % (95 % CI: 6.5, 6.7 %) or 7.5 % (95 % CI: 7.4, 7.6 %) in males and 5.6 % (95 % CI: 5.5, 5.7 %) in females. The prevalence of diabetes was higher in men than in women among all age groups, peaking in the 80–85 year old group (25 %) and in the 85–90 year old group (20 %) for men and women, respectively (Additional file 1: Table S1).

Data availability and the characteristics of the patients are summarized in Table 1. There were strong differences in the number of missing values among the study variables. Virtually all of the patients had age properly recorded, and sex was missing in only 432 (1.3 %) cases. Approximately 75 % of the patients had laboratory results and blood pressure (BP) records. However, BMI, the duration of diabetes, and smoking history were poorly recorded with missing values in 47, 55, and 62 % of the patients, respectively. Cardiometabolic controls were less frequently performed in male and female patients younger than 65 years of age than in older groups. In fact, the proportion of patients with valid data was approximately 10 percentage points lower in this age group than in patients between 65 and 74 years old (Additional file 2: Table S2).

**Table 1:** Data availability, characteristics and clinical parameters of the population of patients with diagnosed diabetes by sex. Navarra (Spain), 2014

	Data available	Total	Men	Women	
Variable	*n* (%)	*n* (%)	*n* (%)	*n* (%)	*p*
Sex	32,206 (98.7)		18,188 (57.47)	14,018 (43.2)	
Age group	32,638 (100)				
<65 years		10,803 (33.1)	7138 (39.2)	3475 (24.8)	
65–74 years		9377 (28.7)	5618 (30.9)	3678 (26.2)	<0.001
≥75 years		12,458 (38.2)	5432 (29.9)	6865 (49.0)	
Treatment	32,638 (100)				
Insulin		6639 (20.3)	3432 (18.9)	3161 (22.5)	<0.001
Oral hypoglycemic		18,551 (56.8)	10,731 (59.0)	7684 (54.8)	
No medication		7448 (22.8)	4025 (22.1)	3173 (22.6)	
Smoking	12,413 (38)	1190 (16.0)	1519 (21.3)	459 (8.8)	<0.001
		Mean (SD)	Mean (SD)	Mean (SD)	
Age (yrs)	32,638 (100)	69.49 (12.82)	67.31 (12.17)	72.4 (13.02)	<0.001
Diabetes duration (yrs)	14,472 (44.3)	10.21 (7.26)	9.81 (7.11)	10.78 (7.45)	<0.001
HbA1c (%)	23,094 (70.8)	7.10 (2.94)	7.07 (3.59)	7.12 (1.83)	0.357
<65 years		7.16 (1.51)	7.16 (1.52)	7.16 (1.52)	0.998
65–74 years		7.02 (2.49)	6.95 (2.89)	7.12 (1.71)	0.006
≥75 years		7.11 (3.94)	7.13 (5.48)	7.10 (2.02)	0.700
Systolic BP (mmHg)	24,616 (75.4)	134.7 (17.3)	134.7 (16.8)	135.0 (17.9)	0.253
Diastolic BP (mmHg)	24,595 (75.4)	75.5 (10.51)	75.87 (10.50)	75.05 (10.45)	<0.001
TGs (mg/dl)	24,381 (74.7)	146.9 (98.4)	146.0 (109.9)	148.1 (81.38)	0.088
HDL (mg/dl)	24,331 (74.5)	45.44 (12.47)	43.35 (11.82)	48.13 (12.74)	<0.001
LDL (mg/dl)	23,891 (73.2)	109.1 (33.8)	106.2 (32.5)	113.8 (34.83)	<0.001
BMI (mg/dl)	17,306 (53.0)	30.47 (9.12)	30.12 (7.95)	30.92 (10.44)	<0.001

The mean age of the patients with type 2 diabetes was 69.5 years, and women were older (72.4 years) than men (67.3 years) (*p* <0.001). The overall duration of diabetes was 10.2 years, and the duration was an average of 1 year longer among women (Table 1).

The average HbA1c level was 7.1 % (64 mmol/mol), both in men and in women; 60.1 % of the patients had HbA1c levels less than 7 % (53 mmol/mol), 82.9 % had HbA1c levels less than 8 % (64 mmol/mol), and 3.4 % had HbA1c levels greater than 10 % (86 mmol/mol) (Tables 1 and 2). The proportion of patients with HbA1c levels less than 7 % (53 mmol/mol) was higher among males (61.2 %) than among females (58.8 %) (*p* <0.001).

**Table 2:** Proportions (%) of patients with type 2 diabetes achieving control goals by sex and age group. Navarra (Spain), 2014

		Men by age group (years)					Women by age group (years)				
Target	Total	All	<65	65–74	≥75	*p*	All	<65	65–74	≥75	*p*
HbA1c ≤7 % (≤53 mmol/mol)	60.1	61.2	58.4	64.4	61.0	0.006	58.8	58.9	58.6	58.8	0.958
HbA1c ≤8 % (≤64 mmol/mol)	82.9	83.5	79.3	86.5	85.1	<0.001	82.2	80.3	83.1	82.5	0.048
HbA1c >10 % (>86 mmol/mol)	3.4	3.4	5.0	2.4	2.5	<0.001	3.5	5.7	2.8	2.8	<0.001
BP ≤130/80 mmHg	39.6	39.5	38.2	37.6	42.6	<0.001	39.6	44.9	39.4	37.6	<0.001
BP ≤140/90 mmHg	69.9	70.0	71.2	69.2	69.8	0.142	69.7	78.1	69.9	66.2	<0.001
LDL ≤100 mg/dl	40.5	44.7	38.6	46.1	49.9	<0.001	35.2	28.9	35.5	37.9	<0.001
HDL (≥35 M, ≥45 W)^a^	69.4	78.3	75.9	80.8	78.2	0.004	58.1	55.9	60.4	57.8	0.365
TGs <150 mg/dl	64.5	66.5	56.1	67.9	76.6	<0.001	62.2	57.9	61.3	64.6	<0.001
BMI <30 kg/m^2^	52.6	54.5	45.3	55.0	63.3	<0.001	50.3	39.2	44.1	59.4	<0.001
Not smoking	84.0	70.4	66.4	80.3	90.3	<0.001	91.2	74.6	92.1	97.8	<0.001
Combined targets:											
A^b^	13.8	16.1	13.0	17.7	17.9	<0.001	11.0	10.2	11.3	11.2	0.236
B ^c^	16.3	18.4	13.0	17.7	25.4	<0.001	13.8	10.2	11.3	16.8	<0.001

^a^Target: ≥35 mg/dL in men, and ≥45 mg/dl in women

^b^A: HbA1c <7 % (53 mmol/mol) & BP <140/90 mmHg & LDL <100 mg/dl

^c^B: HbA1c <7 % (<53 mmol/mol) if <75 years OR HbA1c <8 % (<64 mmol/mol) if ≥75 years & BP <140/90 mmHg & LDL <100 mg/dl

**Fig. 1:**
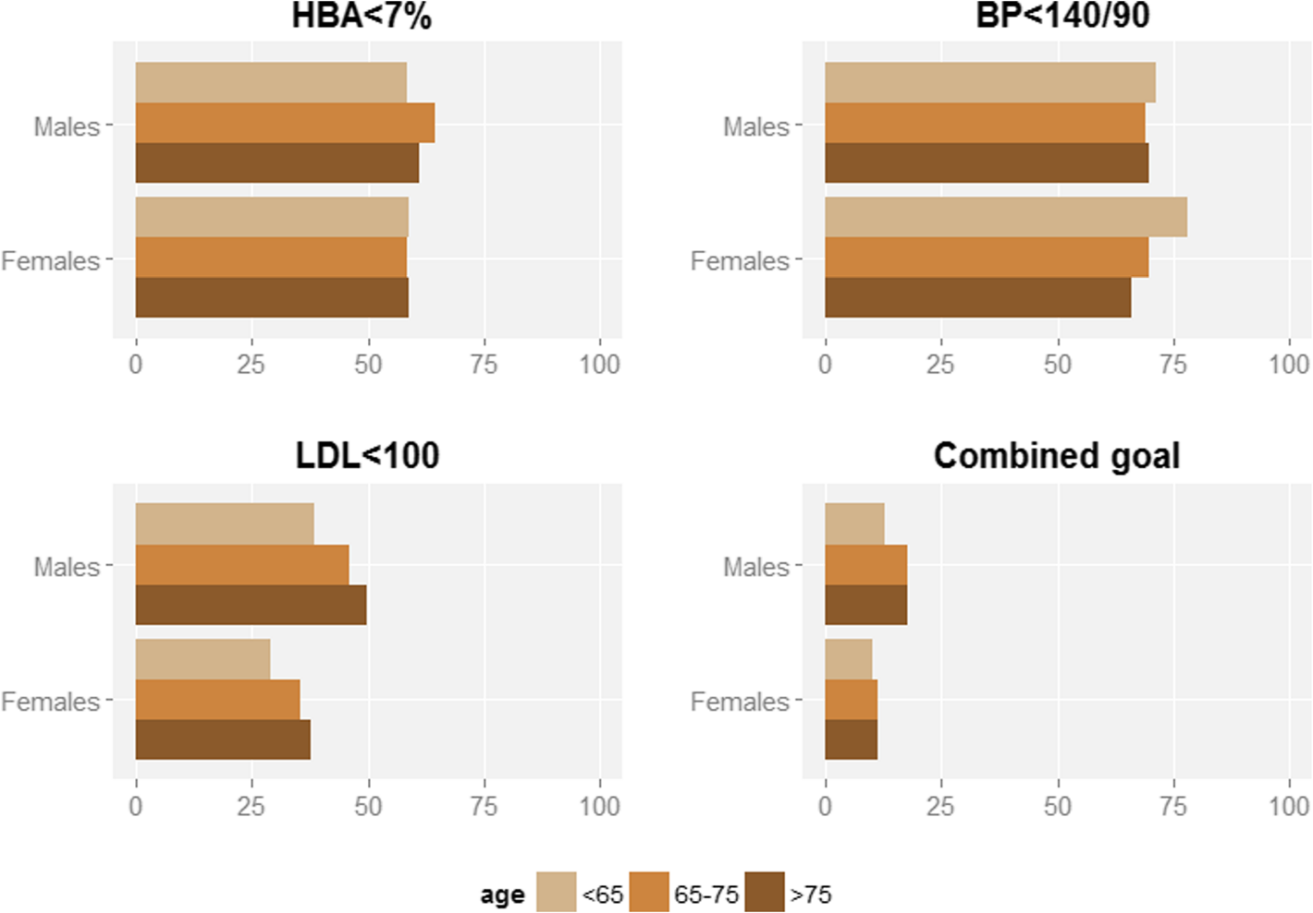
By sex and age group, percentage of patients achieving glycemic and cardiovascular prevention goals. Navarre (Spain), 2014

Adjusting for age, the odds of having HbA1c levels greater than 7 % were 12 % higher among women than among men (OR 1.12, 95 % CI: 1.06–1.19). Similar estimates were obtained after adjusting for age and diabetes duration (OR 1.13, 95 % CI: 1.04–1.24). Some differences between age groups were observed. Among men, the proportion of patients with HbA1c levels less than 7 % (53 mmol/mol) was lower in the group younger than 65 years, whereas among women, there was no significant difference between age groups (*p* = 0.958) (Fig. 1). In both sexes, the group younger than 65 years of age had the highest proportion of patients with an HbA1c level greater than 10 % (86 mmol/mol); this proportion was double the prevalence of all other age groups.

On average, women had slightly lower diastolic BP than men (Table 1), but the control targets were achieved essentially in the same proportions of men and women; 69.9 % of the patients had a BP less than 140/90 mmHg, and 39.6 % had a BP less than 130/80 mmHg (Table 2). No differences between sexes were observed after adjusting for age and diabetes duration.

Overall, 40.5 % of the patients with diabetes had an LDL level less than 100 mg/dl, 69.4 % had an HDL level greater than the target level (35 mg/dl for men, 45 mg/dl for women), and 64.5 % had fasting triglyceride levels less than 150 mg/dl (Table 2). The mean LDL level was 109.1 mg/dl, and it was 7.6 mg/dl higher among women than among men. Men were more likely to meet the LDL control target than women among all age groups, with differences up to 12 percentage points. Adjusting for age, the odds of having an LDL greater than 100 mg/dl was 60 % higher among women than among men (OR 1.60, 95 % CI: 1.52–1.69). Similar estimates were obtained after adjusting for age and diabetes duration (OR 1.58, 95 % CI: 1.46–1.72). Among both males and females, patients under 65 years of age had the poorest LDL control results (*p* <0.001). Regarding HDL, 78 % of males and 58 % of females met the gender-specific targets. Adjusting for age, the men’s odds of having HDL levels greater than 35 mg/dL were 2.65 times higher than the women’s odds of having HDL levels greater than 45 mg/dL (OR 2.65, 95 % CI: 2.50–2.80).

The proportion of patients achieving the composite triple target (simultaneous HbA1c <7 % (53 mmol/mol), BP <140/90 mmHg, and LDL <100 mg/dl; target A in Table 2) dramatically decreased to 14 % (16 % among men and 11 % among women). Among men, the poorest results were achieved in the group younger than 65 years of age (*p* <0.001), whereas among women, no trend was observed across age groups. Using less stringent targets for HbA1c (8 % (64 mmol/mol) in patients over 75 years), the overall proportion of patients achieving the composite control objectives remained low (less than one out of six), and it remained lower in women as well.

Men had lower BMI values than women, and in all age groups the proportion of male patients with a BMI less than 30 kg/m^2^ was greater than that of females. The largest difference in BMI was more than 10 percentage points among the 65–74 year age group. In both men and women, the prevalence of obesity decreased as age increased (*p* <0.001). The prevalence of smoking was lower among women than among men in all age groups (Table 2).

## Discussion

Only a minority of patients with type 2 diabetes met the composite target objectives for glycemic control, blood pressure, and lipid control. Patients under 65 years and women had poorer control of glycaemia and cardiovascular risk factors.

This study had some limitations. First, our analysis was confined to persons with diagnosed diabetes who attended the Regional Heath Service; we had no information about persons using private medical insurance or those with undiagnosed diabetes. Second, there was a risk of information bias from the use of existing electronic clinical records. We cannot rule out that the patients with missing data were different from those with properly recorded data and that they had a different degree of cardiometabolic control. In the case of BMI and smoking status the high proportion of missing values prevents drawing any conclusion about them. The main strengths of this study were that it used data from real medical practice and it included patients from any age, different health status and varying social conditions. Additional strengths may be the large number of data entries available, and the fact that patients who attend the Regional Health Service encompass 96 % of the population in the region. Finally, the sex and age stratified analysis provided an accurate snapshot to identify gaps in the quality of care.

The average levels of HbA1c (7.10 %, 54 mmol/mol) in our population were similar or slightly lower than those from other Spanish regions [13] and European countries [19, 20]. The proportion of patients with good glycemic control (60 %) was higher than the average proportion of the eight European countries (54 %) reported by Stone, but it was within the range of the countries that participated in the study [21]. It was also higher than the proportions found in other Spanish regions several years ago (56 %), in Canada (53 %), and in the USA (52 %) [13, 22–24]. There were some meaningful sex- and age-related differences in glycemic control. Men displayed better glycemic control than women, which is in line with some results previously reported [13, 19], and patients younger than 65 years, particularly males, appeared to have poorer glycemic control than older patients. The highest proportion of patients without a blood test performed in the previous 15 months and the highest proportion of patients with an HbA1c greater than 10 % (>86 mmol/mol) was found in the younger than 65 years age group. Among males, this age group also had the lowest proportion of patients with an HbA1c level less than 7 % (53 mmol/mol). Among women, such a difference was not observed, suggesting that the relationship between age and the achievement of control targets may follow a partially different pattern.

The proportion of patients meeting the blood pressure target of 130/80 mmHg in our study (40 %) was between 8 and 30 percentage points higher than those found in other European countries, but it was 10 percentage points lower than those reported in Canada, and in the USA from the National Health and Nutrition Examination Survey (NANHES) [21, 23, 24]. The low degree of achievement of the 130/80 mmHg target has recurrently prompted the question of whether it is a realistic target for medical practice or whether more feasible targets, such as 140/90 mmHg, should be implemented. However, the better results reported from other populations indicate that further improvements in blood pressure control should be considered feasible in European populations.

Regarding lipid control, the proportion of patients who met the 100 mg/dl target for LDL in Navarre (40.5 %) was similar to those reported from the Spanish studies conducted in other regions and cited above, with differences of less than three percentage points. However, they were 15 percentage points lower than the proportions reported by Stone for eight European countries and the NHANES (2007–2010) for the USA. Additionally, we found poorer achievement of LDL control targets among women and younger patients. Beyond its statistical significance, it was remarkable that the proportion of target-meeting patients was an average of approximately 8 percentage points lower among women than among men and over 10 percentage points lower among patients younger than 65 years than among those older than 75 years. In other words, in terms of risk estimates, a woman’s likelihood of achieving cholesterol targets was notably lower than that of a man.

Only a minority (14 %) of patients met all three control targets for HbA1c, BP, and LDL. Even if less stringent targets are combined, such as 8 % HbA1c (64 mmol/mol) for patients over 75 years, the composite target is only met by less than one-fifth of the patients. Women and patients under 65 years of age were less likely than men and older adults to achieve the composite control targets, following the same patterns observed for LDL and HbA1c. Because early control of the risk factors for microvascular and macrovascular disease may confer benefits, these results suggest that women and younger patients with diabetes require further attention.

There are few studies about the inequalities in cardiometabolic control among patients with diabetes that combine sex and age. Poorer results among younger adults were also found by NHANES (2007–2010) in the USA, in which younger adults were less likely than older adults to meet goals for treatment and preventive practices. In general, sex inequalities were more often reported to be related to cardiovascular risk factors, and poorer glycemic control results were reported to be more related to younger ages, irrespective of the presence of complications [13, 19, 20, 25]. We can only speculate about possible explanations for the observed age and sex-related inequalities in the achievement of the control targets in our population. Because the Regional Health Service provide universal coverage in the community and the same primary care teams provide care to all age groups, we thought that the poorer results of glycemic control among younger patients were more likely related to their personal characteristics, such lifestyle or work activity, rather than to access to health care or the quality of the health care received. Among men older than 65 years, we observed an increase in the proportion of patients who met glycemic targets, but this was not observed in women, which suggests that there may be more significant changes in lifestyles after retirement in men than in women.

In connection with sex-related control inequalities of cardiovascular risk factors, physician attentiveness and selective treatment prescription were suggested as factors that may account for the observed gaps. However, studies conducted in Europe did not support this hypothesis because they did not find therapies to be less stringent among females [22, 25]. More adverse cardiovascular profiles among women and biological differences between men and women have been suggested to explain the greater diabetes-related cardiovascular risk ratios observed among women [26, 27]. Our results support the idea that more adverse cardiovascular profiles among women may play a role.

## Conclusions

This study provides representative snapshots of the degree of cardiometabolic control in a population of patients with diabetes in a Spanish region. Only a minority of patients met the goals of HbA1c, LDL, and blood pressure. Female patients and younger patients were less likely to achieve the control goals. The high prevalence of type 2 diabetes implies that a large portion of the population could benefit from general improvements in control and from reducing age and sex inequalities.

## Additional files

Additional file 1:
**Table S1.** Type 2 diabetes prevalence by age group. Navarre (Spain), 2014 (DOCX 12 kb)
Additional file 2:
**Table S2.** Percentage of patients with at least one measurement recorded in the previous 15 months by age group and sex. Navarre (Spain), 2014 (DOCX 13 kb)
