# Supplementary material for: Sex and age differences in the achievement of control targets in patients with type 2 diabetes: results from a population-based study in a South European region
Source: BMC Fam Pract. 2016 Oct 12;17:144. doi: 10.1186/s12875-016-0533-9 (PMC5060013; doi:10.1186/s12875-016-0533-9)
Supplement: Additional file 1: — Table S1. Type 2 diabetes prevalence by age group. Navarre (Spain), 2014 (DOCX 12 kb) [file 12875_2016_533_MOESM1_ESM.docx]

Supplementary Table 1: Type 2 diabetes prevalence by age group. Navarre (Spain), 2014

| **Age group (yrs)** | **Men (%)** | **Women (%)** |
| --- | --- | --- |
| **(20,24)** | 0.08 | 0.10 |
| **(25,29)** | 0.12 | 0.17 |
| **(30,34)** | 0.34 | 0.29 |
| **(35,39)** | 0.66 | 0.43 |
| **(40,44)** | 1.54 | 0.93 |
| **(45,49)** | 3.06 | 1.58 |
| **(50,54)** | 5.88 | 2.67 |
| **(55,59)** | 9.92 | 4.75 |
| **(60,64)** | 14.42 | 7.01 |
| **(65,69)** | 17.94 | 10.53 |
| **(70,74)** | 23.47 | 15.20 |
| **(75,79)** | 22.41 | 17.64 |
| **(80,84)** | 25.38 | 19.64 |
| **(85,89)** | 22.29 | 20.80 |
| **≥90** | 19.14 | 18.80 |
