# Supplementary material for: Sex and age differences in the achievement of control targets in patients with type 2 diabetes: results from a population-based study in a South European region
Source: BMC Fam Pract. 2016 Oct 12;17:144. doi: 10.1186/s12875-016-0533-9 (PMC5060013; doi:10.1186/s12875-016-0533-9)
Supplement: Additional file 2: — Table S2. Percentage of patients with at least one measurement recorded in the previous 15 months by age group and sex. Navarre (Spain), 2014 (DOCX 13 kb) [file 12875_2016_533_MOESM2_ESM.docx]

Supplementary Table 2. Percentage of patients with at least one measurement recorded in the previous 15 months by age group and sex. Navarre (Spain), 2014

|  | Total (%) |  | Men(%) | | | |  | Women (%) | | | |
| --- | --- | --- | --- | --- | --- | --- | --- | --- | --- | --- | --- |
|  |  |  | <65 yr | 65-74yr | ≥75yr | Total |  | <65 yr | 65-74yr | ≥75yr | Total |
|  | n=32,653 |  | n=7,118 | n=5,628 | n=5,448 | n=18,194 |  | n=3,471 | n=3,671 | n=6,884 | n=14,026 |
| HbA1c checked | 70.7 |  | 64.8 | 75.9 | 73.6 | 70.9 |  | 65.2 | 75.2 | 72.9 | 71.6 |
| Blood pressure | 75.4 |  | 62.8 | 77.6 | 84.2 | 73.8 |  | 66.8 | 80.9 | 83.4 | 78.6 |
| LDL | 73.2 |  | 65.1 | 77.6 | 78.2 | 72.9 |  | 67.0 | 77.7 | 76.8 | 74.6 |
| HDL | 74.5 |  | 67.7 | 78.7 | 79.0 | 74.5 |  | 68.3 | 78.7 | 77.7 | 75.6 |
| Triglycerides | 74.7 |  | 67.9 | 79.0 | 79.0 | 74.6 |  | 68.5 | 78.9 | 77.8 | 75.8 |
| Body Mass Index | 53.0 |  | 45.2 | 58.7 | 58.2 | 53.2 |  | 49.6 | 60.3 | 52.1 | 53.6 |
| Smoking screening | 38.0 |  | 34.5 | 42.4 | 42.2 | 39.2 |  | 33.0 | 37.2 | 39.0 | 37.1 |
